# Retraction: Digital economy, scientific and technological innovation, and high-quality economic development: A mediating effect model based on the spatial perspective

**DOI:** 10.1371/journal.pone.0349728

**Published:** 2026-05-28

**Authors:** The PLOS One Editors

The *PLOS One* Editors retract this article [1] because it was identified as one of a series of submissions for which we have concerns about compromised peer review.

The Editors have no evidence of any author involvement in the peer review concerns.

YL did not agree with the retraction. SZ either did not respond directly or could not be reached.
